# Intercalary Tricortical Iliac Crest Graft for Treatment of Midclavicle Nonunion With Bone Loss: Two Case Reports and Review of Literature

**DOI:** 10.7759/cureus.40265

**Published:** 2023-06-11

**Authors:** Austin Williams, Mohammed Miniato, Adam Pasquinelly, Marshall Gillette, Christopher Sanford, Matthew Graves

**Affiliations:** 1 Department of Orthopedic Surgery, The University of Toledo College of Medicine and Life Sciences, Toledo, USA; 2 Department of Anesthesiology, Baylor College of Medicine, Houston, USA; 3 Department of Orthopedic Surgery, University of Mississippi Medical Center, Jackson, USA

**Keywords:** fracture repair bone graft, iliac crest bone graft, nonunion fracture, clavicle reconstruction, clavicle

## Abstract

Several articles support the use of cancellous iliac crest bone grafting in the treatment of clavicle nonunion; however, there is very little literature on the use of tricortical iliac crest grafts in the setting of clavicle nonunion with bone loss. When it has been studied, tricortical grafting has been shown to produce radiologically confirmed union in the clavicle, leaving patients satisfied with the ultimate outcome. We present two cases of clavicle fracture nonunion successfully treated with tricortical interposition bone grafting. In the first case, a 45-year-old female presented with an atrophic left midshaft clavicle fracture nonunion with failed hardware that had undergone two previous attempts at fixation without achieving union. She was treated with a structural interposition iliac crest bone graft with plate fixation and regained full painless function of the arm with radiographic fracture union. In the second case, a 50-year-old male presented after a left midshaft clavicle fracture that had undergone acute stabilization, followed by revision for nonunion that was unsuccessful, resulting in persistent nonunion with bone loss. He was treated with a tricortical iliac crest bone graft and plate fixation. Cultures from the time of surgery did grow *Staphylococcus epidermidis *and *Propionibacterium acnes,* and he was treated with intravenous vancomycin for six weeks. The patient’s clavicle went on to union and he regained full, painless function by his six-month follow-up visit. These cases demonstrate the use of tricortical interposition bone grafting with compression plating as a viable option for rare instances in which previous surgical intervention has failed to progress a midshaft clavicle fracture to union.

## Introduction

Clavicle fractures are relatively common and represent around 2-5% of all fractures, with children and young adults being the most affected groups [1,2]. About 75-80% of clavicular fractures occur in the middle third of the clavicle [3]. Non-surgical treatment of clavicular fractures has been shown to have up to a 15% rate of nonunion, particularly with initial shortening greater than 2 cm [4]. Following open reduction and internal fixation (ORIF), however, the rate of nonunion decreases to less than 1% making nonunion of the clavicle a relatively uncommon occurrence [5]. A variety of techniques have been described for the treatment of nonunion after primary osteosyntheses with successful results [6]. However, when initial attempts at treatment of nonunion fail and are associated with significant bone loss, there is much less literature available [7-9]. A single recent study was identified that focused on tricortical iliac crest grafts for clavicle fracture nonunion, with a sample size of only five cases, that demonstrated the successful use of tricortical iliac crest grafts in large clavicle nonunion defects rather than vascularized grafts from other locations [10]. To add to this very small dataset, we present two cases of clavicle fractures that failed both initial osteosynthesis and an attempted revision procedure. These cases were complicated by significant bone loss and shortening that were successfully treated with a structural tricortical iliac crest bone graft and revision fixation.

## Case presentation

Case 1: History

**Figure 1:**
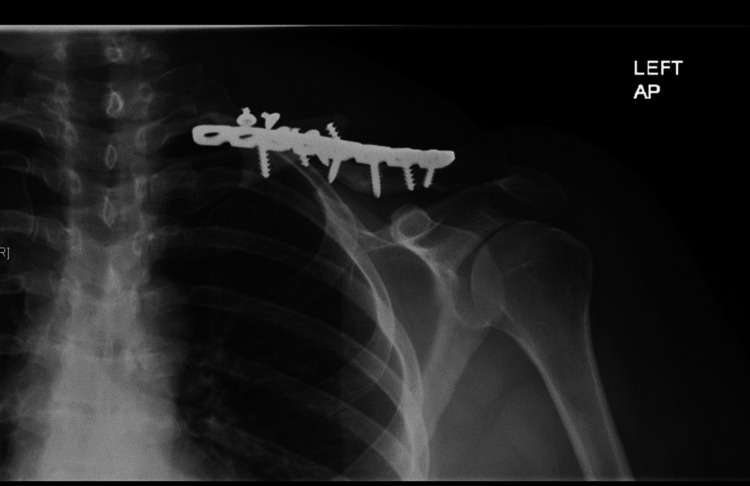
Pre-operative anteroposterior x-ray of the left clavicle showing hardware failure and nonunion.

A 45-year-old Caucasian female initially sustained a left, midshaft clavicular fracture ten years prior to presentation. She underwent open reduction and internal fixation (ORIF) at the time of the initial injury. She went on to develop fracture nonunion and her hardware subsequently failed. She then underwent revision surgery that included a cancellous iliac crest autograft with revision plate fixation five years after the index procedure. She reported an initial improvement in pain after this surgery; however, her pain and functional limitations returned over the next few years. She presented to our clinic a total of five years after her revision surgery with severe left shoulder pain and disability. She reported no interval trauma to the extremity. On examination, the incision site from the previous operations was well-healed and showed no signs suggestive of infection. There was tenderness and crepitus on palpation of the clavicle. The infectious workup was negative, with serum white blood cell count, erythrocyte sedimentation rate, and C-reactive protein within normal limits. X-rays obtained at the time of presentation demonstrated an atrophic left clavicle fracture nonunion with failed hardware and shortening of the clavicle (Figure 1). Given the history, radiographic findings, and persistent pain and disability, we offered the patient left clavicle hardware removal, nonunion takedown, and tricortical iliac crest bone grafting with revision ORIF.

Case 1: Surgical technique

**Figure 2:**
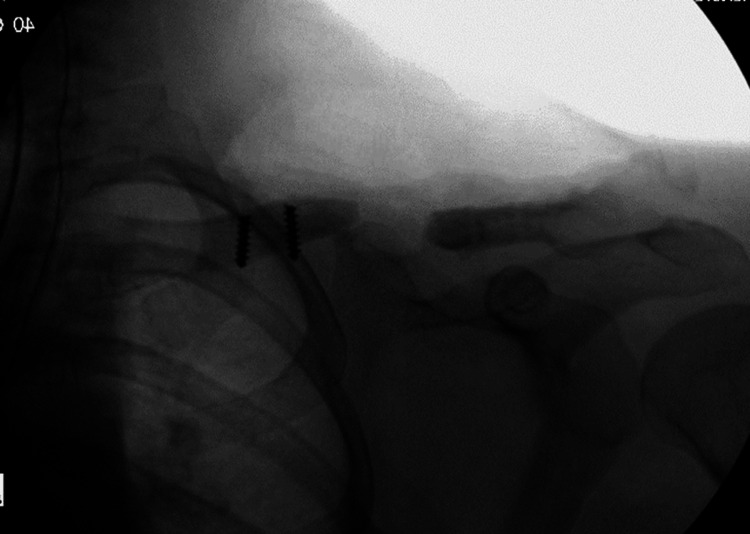
Intraoperative fluoroscopy showing removal of failed hardware and debridement of atrophic bone (Note: two broken screws were retained within the medial portion of the clavicle).

The prior surgical incision was utilized, taking care to preserve branches of the supraclavicular nerve. The failed hardware was readily identified and removed. The ends of two broken screws buried well within the medial segment were retained (Figure 2).

There was no obvious evidence of infection, but multiple cultures were obtained from the nonunion site and surrounding soft tissue to identify any indolent infectious etiology. The fracture ends were exposed and noted to be sclerotic. These were debrided until punctate bleeding was obtained on each side of the fracture. The medullary canal on each side of the fracture was accessed with a drill bit to increase vascularity as well as prepare the clavicle to accept the intercalary graft. After preparing the nonunion site and removing the sclerotic bone, the gap between the fracture ends measured approximately 3 cm.

**Figure 3:**
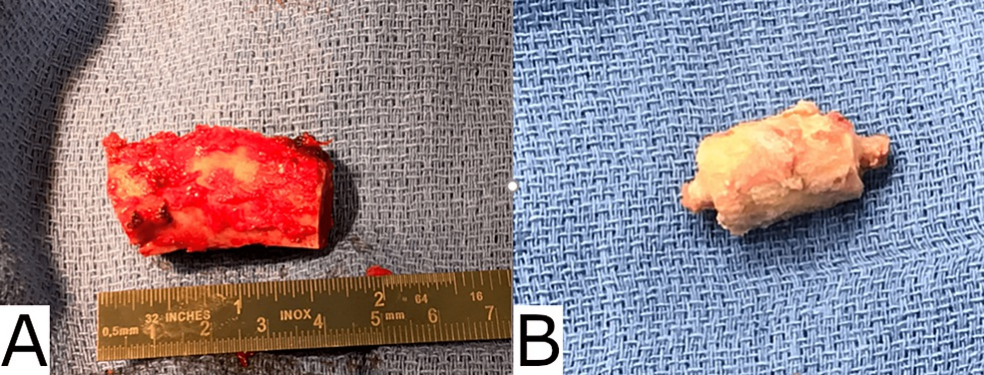
The tricortical graft was taken from the left iliac crest (panel A) and contoured to form pegs on either end (panel B).

An appropriately sized tricortical graft from the ipsilateral iliac crest was harvested approximately three fingerbreadths posterior to the anterior superior iliac spine (ASIS) using an oscillating saw and osteotome (Figure 3A). Care was taken to leave a large segment of the ASIS bone distally and to not make an undercut in this area in order to prevent the possibility of a future fracture. Cancellous iliac crest bone graft was also harvested. A sagittal saw and burr were used to shape the graft to fit the defect with pegs to hold the graft in place, facilitate integration, and interdigitate with the clavicle on either side (Figure 3B).

**Figure 4:**
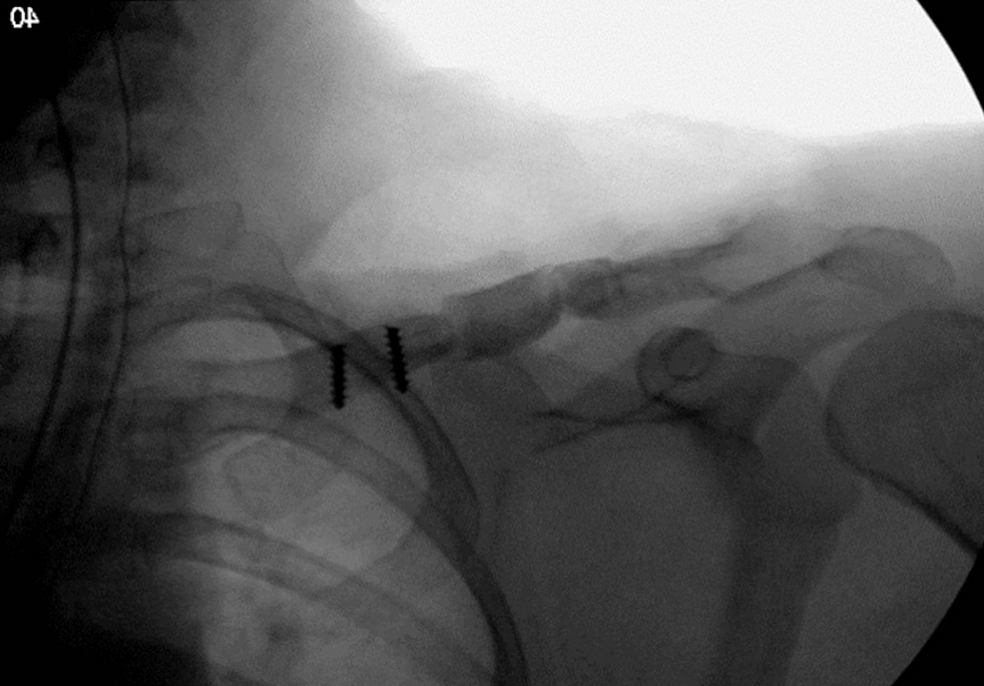
Intraoperative fluoroscopy showing placement of the structural iliac crest graft.

This graft was placed in the defect with the cortical face of the graft positioned caudally on the compression side of the clavicle. The pegs were inserted into the medial and lateral ends of the clavicle. Fluoroscopy demonstrated appropriate positioning of the graft with restored length (Figure 4).

**Figure 5:**
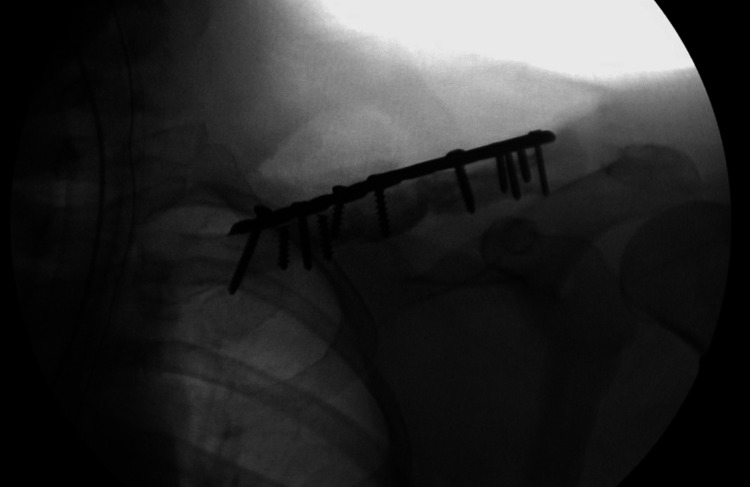
Intraoperative fluoroscopy showing graft placement and the complete construct.

A 7-hole 3.5 mm plate with a lateral locking cluster was then placed superiorly, and compression of the graft was obtained through the plate with eccentric drilling. Additional screws were added to obtain a stable construct, including a screw to stabilize the intercalary graft (Figure 5). The wound was irrigated, and the remaining cancellous bone graft harvested from the iliac crest was packed around the nonunion site, followed by standard closure.

Case 1: Postoperative course

**Figure 6:**
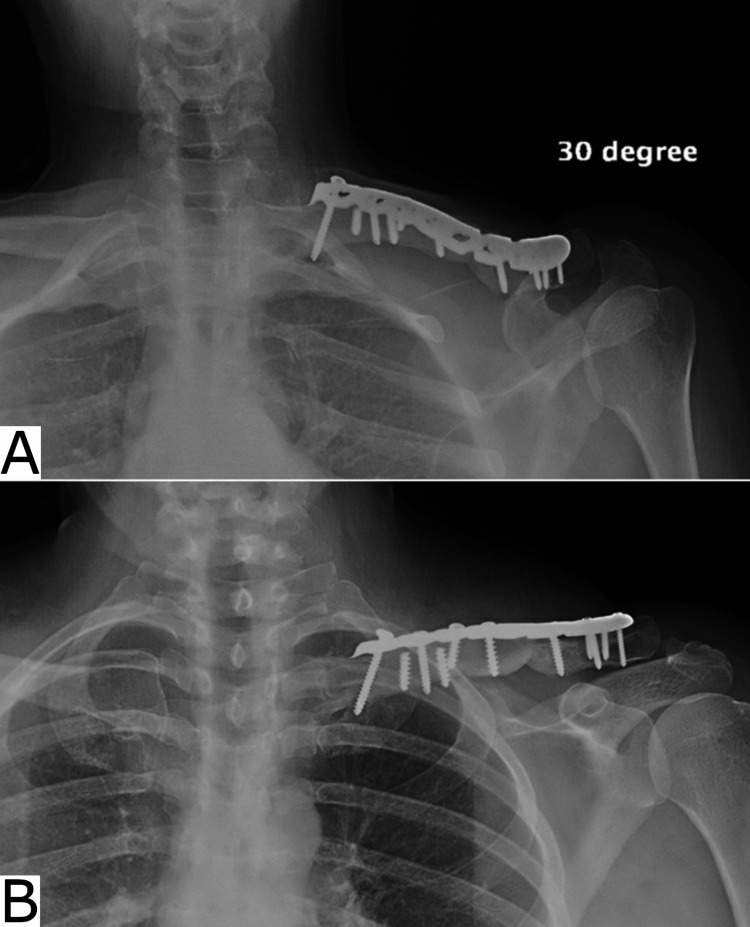
Eight-month postoperative radiograph taken from 30 degrees below the clavicle (panel A) and direct anteroposterior (panel B) showing bony bridging and complete healing of the graft.

The patient was discharged in a simple sling and was scheduled for clinical follow-up with interval radiographs. At two weeks postoperatively, she began pendulum exercises and passive range of motion with physical therapy guidance. The patient gradually increased her activity, and at her last follow-up, she was using the extremity without limitations. Clinical exam demonstrated no pain with full shoulder range of motion and strength equal to the contralateral upper extremity. Radiographs showed complete healing and incorporation of the graft (Figure 6).

Case 2: History

**Figure 7:**
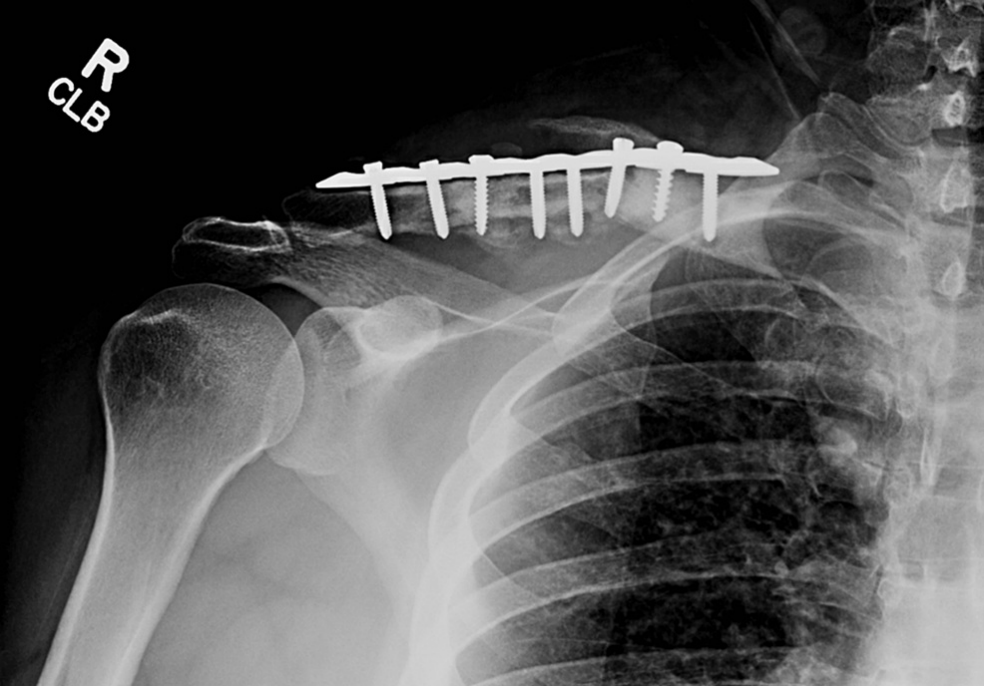
AP radiograph of the right clavicle demonstrating atrophic nonunion, bony erosions, and hardware failure.

A 50-year-old Caucasian male sustained a right midshaft clavicle fracture one year prior to the presentation that was initially treated with acute ORIF by an outside surgeon, followed by revision ORIF four months later for nonunion, at which time an allograft and bone morphogenic protein graft was used. However, this failed to promote union, and the patient had persistent pain as well as prominent hardware. Imaging obtained upon presentation demonstrated atrophic nonunion of the clavicle fracture and loosening of the hardware with some bony erosions (Figure 7). Examination of the patient’s shoulder revealed a well-healed incision without any evidence of infection. There was pain with any attempted range of motion of the shoulder. The infectious workup was negative, with serum white blood cell count, erythrocyte sedimentation rate, and C-reactive protein within normal limits. The patient was offered revision ORIF with intercalary tricortical iliac crest bone grafting.

Case 2: Surgical technique

**Figure 8:**
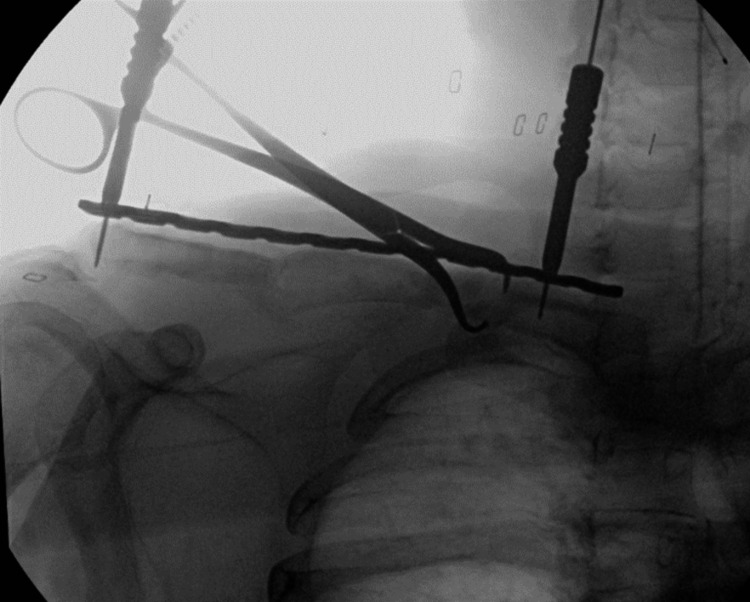
Intraoperative fluoroscopic imaging showing placement of an intercalary iliac crest bone graft.

The patient’s previous surgical incision was utilized. No evidence of infection was noted, but multiple cultures were obtained due to the recalcitrant nonunion. A Gram stain performed during the operation did not reveal any organisms. The clavicular nonunion was taken down, and the tricortical graft was harvested, fashioned, and placed in the same manner as in the prior case (Figure 8). Due to poor bone quality, a 3.5-mm reconstruction plate was placed on the superior surface of the clavicle to span the bone from the acromioclavicular joint to the sternoclavicular joint. The graft was compressed between the two main segments of the clavicle using nonlocking screws, followed by locking screws. In order to empower this fixation, a 3.5-mm locking compression plate was contoured to fit the anterior aspect of the clavicle and secured. Irrigation was performed, followed by placing the remainder of the cancellous iliac crest autograft along with the demineralized bone matrix around both junctions of the intercalary graft. Vancomycin powder was placed into the wound, and a layered closure was performed.

Case 2: Postoperative course

**Figure 9:**
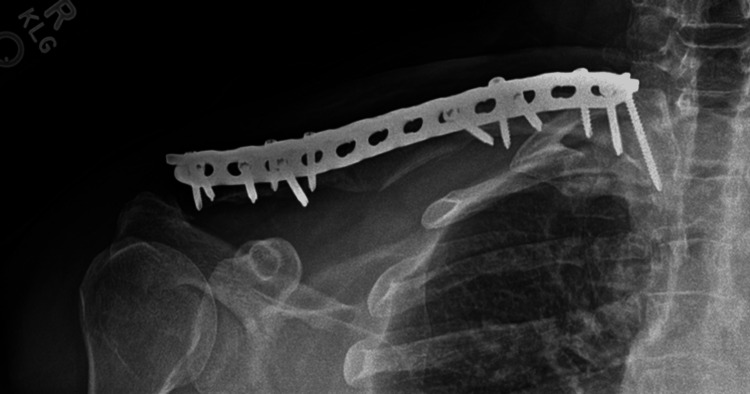
Six-month postoperative radiograph showing the integration of the bone graft and fracture union.

Despite a negative intraoperative Gram stain, cultures grew *Staphylococcus epidermidis *and *Propionibacterium acnes*. Infectious disease was consulted, and the patient underwent a six-week course of intravenous vancomycin. Three weeks following surgery, the patient developed a small seroma beneath his incision. Due to his positive cultures, this was evacuated with irrigation and debridement. There was no evidence of purulence or infection upon evacuation, and cultures taken at that time were negative. The patient completed his course of antibiotics and progressed with postoperative rehabilitation. At his last clinical follow-up, his incision was well healed, the patient had full, painless motion of the shoulder, and imaging demonstrated a successful union of the clavicle fracture (Figure 9).

## Discussion

Nonunion is defined as a fracture that is unlikely to progress to union without further intervention [11]. Several factors can lead to nonunion, including open method of reduction, open fracture, presence of post-surgical fracture diastasis, infection, comminuted fracture pattern, high degree of initial fracture displacement, lack of adequate mechanical stability, poor blood supply, lack of soft tissue coverage, and the location of the fracture [12]. Patient-modifiable risk factors such as smoking, alcohol use, diabetes, and nonsteroidal anti-inflammatory drug (NSAID) use have also been identified and should be optimized when managing patients with nonunions [12]. Identification of a nonunion begins with a thorough history of the fracture and prior treatment alongside radiographic analysis. Nonunion can be described as septic, pseudoarthrosis, hypertrophic, atrophic, or oligotrophic. A study by Gausden et al. reported on a series of clavicular nonunion of which 83% of failed primary osteosyntheses returned positive cultures for *Propionibacterium acnes* [13]. These were treated with revision fixation and culture-sensitive antibiotics in a single-stage procedure that resulted in a 100% rate of union. Based on this study, the authors recommend intraoperative cultures when treating any nonunion to search for an infectious cause of the nonunion, even when there is low clinical suspicion.

Clavicle nonunion is rare, even with conservative treatment. Nonunion is even less common in fractures undergoing internal fixation [5]. Several techniques can be used to treat a nonunion, and the indications for each depends on the etiology and degree of bone loss. Revision open reduction and internal fixation with superior plating, bone stimulation, cancellous bone grafting, and the use of bone morphogenic protein and osteoconductive agents are well-studied treatment options that have shown positive outcomes [2,14]. In atrophic nonunion, such as the cases presented, the inadequate blood supply to the fracture site and poor biology can lead to sclerotic, non-healing bone ends that can often be seen on radiographs. Surgical decortication until the bleeding bone is obtained and opening of the medullary canal on both sides of the fracture will allow entry of progenitor cells to the fracture site that will aid in healing. During this process, care should be taken not to shorten the clavicle by more than 1 cm compared to the uninjured side, as this may cause glenohumeral and/or scapulothoracic joint dysfunction [13]. When there is shortening of the clavicle, such as in the presented cases, a structural graft from the iliac crest may be taken and the lateral and medial edges sculpted into pegs that can be inserted into the prepared medullary canals. This structural graft fills the nonunion and returns the clavicle back to the desired length. The cortical section of the graft is placed inferiorly to create a smooth surface and avoid any excessive callus formation that may cause neurovascular impingement. This placement also biomechanically strengthens the clavicle as it is fixed with a superiorly placed plate construct [14]. The use of tricortical grafts can provide even more stability in the construct [15,16].

## Conclusions

In this case series, two patients presented with midshaft clavicle fracture nonunions with failed hardware that had each undergone previous attempts at fixation without achieving union. They were treated with a structural interposition iliac crest bone graft with plate fixation and regained full painless function of the arm with radiographic fracture union. Several articles support the use of cancellous iliac crest bone grafting in the treatment of clavicle nonunion, though there is very little literature on the use of tricortical iliac crest grafts in the setting of clavicle nonunion with bone loss. When it has been studied, tricortical grafting has been shown to produce radiologically confirmed union in the clavicle leaving patients satisfied with the ultimate outcome. Tricortical interposition bone grafting with compression plating is a viable option for the rare instances in which previous surgical intervention has failed to progress a midshaft clavicle fracture to union.
